# Inflammation profiles in Alzheimer's disease relate to cognition and neurodegeneration

**DOI:** 10.1002/alz.71642

**Published:** 2026-06-27

**Authors:** Katherine R. Birditt, Kalliopi Mavromati, Peter Swann, Terence Quinn, Atticus H. Hainsworth, Lynne Hughes, John T. O'Brien, William McEwan, Maura Malpetti

**Affiliations:** ^1^ Department of Clinical Neurosciences University of Cambridge and Cambridge University Hospitals NHS Trust, University of Cambridge Cambridge UK; ^2^ UK Dementia Research Institute at University of Cambridge Cambridge UK; ^3^ School of Cardiovascular and Metabolic Health University of Glasgow Glasgow UK; ^4^ Department of Psychiatry, School of Clinical Medicine University of Cambridge Cambridge UK; ^5^ Molecular and Clinical Sciences Research Institute St George's University of London London UK; ^6^ Department of Neurology St George's University Hospitals NHS Foundation Trust London UK; ^7^ Global Alzheimer's Platform Foundation Washington, Washington DC USA

**Keywords:** Alzheimer's disease, blood‐based biomarkers, cytokines, neuroinflammation, neurodegenerative diseases, multiplex assays

## Abstract

**INTRODUCTION:**

Immune signaling alterations have been implicated in Alzheimer's disease (AD) pathophysiology, but their heterogeneity across the disease continuum in real‐world cohorts is poorly characterized, limiting the development of stratified immunomodulatory approaches.

**METHODS:**

In a diverse multicenter cohort (BioHermes) of 176 amyloid‐positive individuals with AD/mild cognitive impairment (MCI) and 173 age and sex‐matched controls, principal component analysis was performed on Luminex‐measured plasma cytokines to derive inflammatory signatures, and their direct/indirect associations with cognition and neurodegeneration.

**RESULTS:**

Two components were identified. Proinflammatory Component 2 was elevated in AD/MCI and in Black/African American participants, and strongly associated with poorer cognition (independently of neurofilament light [NfL], phosphorylated tau 217 [p‐tau217], and glial fibrillary acidic protein [GFAP]). Inflammatory Component 1 showed an indirect association with cognition, mediated by neurodegeneration (plasma NfL).

**DISCUSSION:**

Plasma inflammation profiles were associated with poorer cognition via direct and neurodegeneration‐mediated pathways, supporting their potential use as stratification markers in AD therapeutics.

## Highlights

- Data‐driven cytokine profiles differ between patients with Alzheimer's disease and healthy participants.
- Pro‐inflammatory signatures are elevated in Black/African American individuals relative to White and Asian participants.
- Plasma phosphorylated tau 217 (p‐tau217) is not associated with inflammatory signatures.
- Neurofilament light (NfL) mediates pro‐inflammatory profile's effects on cognition.

## 1 BACKGROUND

Alzheimer's disease (AD) is a neurodegenerative disorder characterized by extracellular amyloid beta (Aβ) plaques and intracellular tau neurofibrillary tangles.^1^ In addition to these hallmark pathological features, chronic neuroinflammation has also emerged as an important contributor to disease pathogenesis and progression.^2^, ^3^, ^4^ Postmortem studies have found that activated microglia co‐localize with Aβ plaques and tau tangles.^5^ Sustained glial responses disrupt the modulatory balance of pro‐ and anti‐inflammatory cytokines, potentially exacerbating tau phosphorylation and aggregation and contributing to the self‐perpetuating cycle of neuronal injury.^6^, ^7^ Cytokines play a role in initiating and modulating the immune response through autocrine, paracrine, and endocrine regulatory effects.^2^, ^8^ Although there is inconsistent evidence on blood cytokine levels in AD, with some markers increased in certain cohorts and unchanged or reduced in others, several studies identified that levels of interleukin‐6 (IL‐6), soluble tumor necrosis factor receptor 1 (TNFR‐I) and receptor 2 (TNFR‐II), IL‐1β, and C‐reactive protein (CRP) are consistently increased in patients with AD compared to controls.^8^

Studies have found that peripheral measures of inflammation, including cytokines and peripheral blood mononuclear immune cells, are related to central brain inflammation levels, pathology, and faster clinical decline across neurodegenerative diseases.^9^, ^10^, ^11^ Neuroinflammation, measured by positron emission tomography (PET) with tracers targeting the translocator protein tracers (TSPO), has been shown to associate with faster tau accumulation and, in some cases, to precede it.^4^ Elevated pro‐inflammatory cytokines in plasma have been associated with poorer cognitive performance in individuals with AD, an effect that could be induced by microglial activation and accelerated neuronal injury.^12^, ^13^, ^14^ Whether peripheral inflammation influences cognition directly or through intermediate neurodegenerative processes remains unresolved in AD.^12^

Disentangling the effects of inflammation, neurodegeneration, and amyloid/tau (A/T) pathology on clinical outcomes is challenging. Path analysis, within a structural equation modeling (SEM) framework, offers a solution by simultaneously estimating the direct and indirect pathways between variables.^15^ Prior SEM applications in AD have demonstrated direct impacts of tau pathology on cognition and indirect effects via atrophy, parsing the relative contributions of multiple pathological markers to prediction of cognitive decline.^3^, ^16^, ^17^ Applying this framework to inflammatory, neuropathological, and cognitive measures can help to clarify whether inflammation exerts an independent effect on cognition or operates through the mediation effects of neurodegeneration. Inflammation's effects on clinical outcomes have been suggested to be partially mediated by neuroaxonal damage,^13^ which can be proxied by plasma neurofilament light (NfL) levels. However, inflammation could also impact cognition by other pathways, including modulation of vascular factors, synaptic dysfunction, and neurotransmitter changes,^2^ which cannot be captured by neuroaxonal damage measurements.

Of note, most studies investigating inflammatory signatures in patients with AD have been conducted primarily in non‐Hispanic White populations. Emerging literature exploring racial disparities in AD mirrors broader epidemiological evidence of ancestral differences in systemic inflammation.^18^, ^19^ A recent, non–dementia‐specific meta‐analysis identified marked racial differences in inflammation blood markers, suggesting a complexity of inflammatory profile differences between Black and White individuals.^19^, ^20^ Given that peripheral inflammatory biomarkers are influenced by ethnicity, relying on signatures derived primarily from non‑Hispanic White cohorts may lead to poorly generalizable findings, reduced diagnostic sensitivity, or overlooked risk pathways in under‑represented groups. Therefore, characterizing distinctive inflammatory plasma profiles in AD cohorts that include patients from different ethnicities can provide further insights into potential differential risk pathways and ensure the generalizability of biomarker findings.

In this study, we aimed to identify blood‐based inflammation profiles in a large, population‐representative cohort of patients with AD, including participants from a non‐White background. Specifically, we (1) identified inflammatory signatures that differ between people on the AD spectrum and cognitively unimpaired volunteers; (2) characterized their relationships with pathology and neurodegeneration biomarkers; and (iii) employed mediation path analysis to parse direct versus indirect pathways linking inflammation, neurodegeneration, and cognitive outcomes.

**RESEARCH IN CONTEXT**

1. **Systematic review**: Altered immune signaling is recognised as integral to Alzheimer's disease (AD) pathophysiology, yet most plasma cytokine studies have focused on single markers in relatively homogeneous cohorts, thereby limiting insights into coordinated inflammatory patterns and their variation across ethnically diverse populations.
2. **Interpretation**: In the Global Alzheimer's Platform Foundation's Bio‐Hermes data, we profiled plasma cytokines in amyloid‐positive participants with mild cognitive impairment and AD dementia and amyloid‐negative controls, across multiple ethnic backgrounds. Principal component analysis revealed two data‐driven inflammatory components that showed distinct associations with neurofilament light and Mini‐Mental State Examination scores, indicating that coordinated immune signatures track core neurodegenerative pathology and cognitive status.
3. **Future directions**: Replication in independent and longitudinal cohorts incorporating imaging endpoints is required to test temporal ordering, prognostic value, and clinical utility. Future work should also account for the influence of comorbidities and polypharmacy, and disentangle AD‐specific immune signatures from inflammation related to aging, vascular pathology, and infections.

## 2 METHODS

### 2.1 Participants

For our study, we leveraged the Global Alzheimer's Platform Bio‐Hermes Dataset, which had recruited ethnically diverse and community‐based participants from 17 sites across the United States.^21^ Prior to any study procedures, informed consent was obtained from all participants. The protocol was reviewed and approved by Advarra, a central institutional review board (Reference Number Pro00046018). The study is registered on ClinicalTrials.gov (NCT04733989) and was conducted in accordance with the ethical standards as laid down in the 1964 Declaration of Helsinki and its later amendments. To be included in this study, individuals had to have a normal Geriatric Depression Scale (GDS) score, no 12‐month history of stroke or seizures and no history of cancer within the past 5 years (excluding melanoma skin, or prostate cancer in situ).^21^ Those with unstable medical conditions that could affect cognitive assessments or conditions that would rule out participation in a therapeutic trial were excluded, along with individuals on immunosuppressive medication. Amyloid positivity/negativity were defined based on amyloid PET imaging, with central visual reads of florbetapir (Amyvid) PET scans per the manufacturer's criteria, or at cerebrospinal fluid (CSF) Aβ42/Aβ40 levels.^21^ Cognitive performance was assessed using Mini‐Mental State Examination (MMSE) scores (0–30 scale), treated as a continuous variable.^22^ Cognitively unimpaired participants were defined by MMSE >26–30. All participants provided written informed consent, with the study approved by appropriate institutional review boards at each site. Ethnicity was defined as self‐reported ancestral backgrounds.

### 2.2 Plasma biomarker measurements

Whole‐blood samples were processed for plasma separation and stored at –80°C within 4 h of blood draw. Plasma cytokine levels were quantified with the 65‐Plex Luminex xMAP immunoassay (Merck Millipore). Twenty‐eight of 65 analytes were excluded as they showed low detectability (>75% missingness across patients and controls) (Table S1). To exclude variables that would compromise reliable modeling, plasma proteomics analyses have previously applied a 70%–75% missingness cutoff to filter proteins before differential testing in studies on AD.^23^ We also compared this cutoff to a threshold of >50% missingness to see if the patterns of cytokine variation did not change using a more conservative cutoff (Figure S1). For the remaining 37 markers, missing values were imputed via Multivariate Imputation by Chained Equations (MICE) before statistical analyses. This method of imputation was selected because, although it assumes missing at random, it leverages correlations across biomarker and demographic information to generate plausible values, thereby reducing bias and loss of power compared with simpler methods such as mean substitution or complete‐case analysis.^24^ Each cytokine variable was then log_10_‐transformed, adjusted for age and sex, by fitting a linear regression model from which the residuals were extracted, and scaled prior to principal component analysis (PCA). Additional available plasma biomarkers reflecting AD pathology and neurodegeneration included levels of phosphorylated tau 217 (p‑tau217) (in‑house Simoa assay).^25^ NfL and glial fibrillary acidic protein (GFAP) quantified by a Quanterix Simoa 4‑Plex. These three markers were also log‐transformed prior to analysis.^26^

### 2.3 Statistical analysis

All analyses were performed R (v4.3.1) in RStudio hosted on the Alzheimer Disease Data Initiative (ADDI) online platform (https://www.alzheimersdata.org). PCA was applied to the cytokine variables to reduce the dimensionality of the data and identify underlying patterns of covariation.^27^ An orthogonal varimax rotation was applied to produce uncorrelated components, facilitating the interpretability of each component as an inflammatory signature loaded onto different cytokines. The number of components to retain was based on eigenvalues >1 using default Kaiser criterion and ≈10% variance explained by each component.^28^ Outlier detection was applied to identify cytokine values that may have arisen from sample issues or acute systemic inflammatory conditions that could impact the component results. Mahalanobis distance (MD) was calculated for each component on individual scores to identify multivariate outliers.^29^ Subjects with an MD exceeding the chi‐square threshold at the 97.5th percentile (degrees of freedom [*df*] = 2*; χ^2^* = 7.378, MD >2.72) were considered outliers and removed from subsequent analyses (Figure S2). The varimax rotated PCA was then re‐run following outlier removal.

Next, the resulting component scores were compared between patients with AD and healthy participants using non‐parametric Wilcoxon rank‐sum tests. Given the diversity of the recruited population, we also explored whether the cytokine profiles differed across these self‐defined ethnic groups with a Kruskal–Wallis test. To test for any confounding effects of ethnicity on group differences in the component scores, we also ran a linear model with both ethnicity and groups as predictors of component scores.

Associations between inflammation components, plasma pathology markers, and MMSE were tested via robust regression using an M‑estimator with Huber weighting to reduce the influence of outliers and provide reliable estimates even when error distributions deviate from normal.^30^ Age and sex were included in all regression models as covariates to account for their effect on plasma pathology markers and cognitive scores. Benjamini–Hochberg False Discovery rate (BH‐FDR) (*q* = 0.05) corrections were applied to each component in relation to the three plasma models, and the correlations with MMSE separately, to account for multiple comparisons.

Guided by an a priori two‐path hypothesis that inflammation influences clinical outcomes (i.e., cognitive impairments) via (1) a neurodegeneration‐mediated pathway and (2) a direct, neurodegeneration‐independent pathway, we pre‐specified a mediation path analysis using the R *lavaan* package to test this. This framework was used to evaluate whether the data were consistent with a theoretically motivated structure rather than to estimate causal effects. In the model, we focused on NfL over other plasma markers, as a proxy of neuroaxonal injury.^31^, ^32^ We estimated a full path model linking both Component 1 and Component 2 scores, through direct and indirect (mediated by NfL) paths, to MMSE. Age and sex were included as covariates. Point estimates were obtained by maximum likelihood (ML), with robust Huber–White standard errors and Yuan–Bentler scaled tests for inference and model selection. PC scores were entered as observed exogenous variables. Their pairwise covariances were fixed to zero, preserving the sample orthogonality of the components in the model.

We first fit the full model, and we then applied a stepwise selection, testing with a Wald test whether the least significant path should be removed from the model to improve the fit. We then compared the full and reduced model with Akaike inclusion criteria (AIC).^33^ If the Wald test was non‐significant and AIC improved, we fixed the tested path to zero and refit the model. We pre‐specified that all statistically significant paths would be retained and not subject to Wald testing. For interval estimation in the reduced model, we used a nonparametric bias‐corrected and accelerated (BCa) bootstrap under the ML estimator. We generated 4000 bootstrap samples by case resampling, refit the same model to each sample, and computed 95% BCa confidence intervals (CIs) from the resulting bootstrap distribution of each parameter. The model had goodness‐of‐fit indices—chi‐square test, Comparative Fit Index (CFI), Tucker Lewis Index (TLI), and root mean square error of approximation (RMSEA)—extracted.^34^

## 3 RESULTS

### 3.1 Participant characteristics

We initially included 349 participants in the study, but following outlier removal within PCA, the final sample size was reduced to 337 individuals (171 AD/mild cognitive impairment [MCI] and 166 controls; 10 Asian, 29 Black/African American, and 298 White individuals) (see Table 1 for final cohort). The patient group scored significantly worse on cognitive scores than controls. Approximately 11.5% of the cohort self‐identified as Black/African American and 3.5% as Asian, and there was a similar proportion of patient and control subgroups across the three different ethnicities as illustrated by the non‐significant Fischer's exact test (*p* > 0.61). All Wilcoxon rank‐sum tests confirmed significant differences between plasma biomarkers. Clinical and biological differences by ethnicity were reported (Table S2). Medical history reports showed a similar mean and standard deviation (SD) of past health‐related conditions and comorbidities between patients (number of conditions, mean ± SD: 2.12 ± 1.20) and controls (mean ± SD: 2.34 ± 1.32). However, the number of health‐related conditions covered several categories, which made it difficult to systematically correct for them in our analyses (see Figure S3 for prevalences of conditions by category and groups).

**TABLE 1:** Demographics, clinical, and biological information shown by group.

Variable	AD/MCI	Control	*p*‐value
*N*	171	166	
Age, mean ± SD	75.0 ± 5.8	74.2 ± 6.0	0.2128
Sex, F / M	83 / 88	79 / 87	0.9481
Ethnicity–Asian	5	5	
Ethnicity–Black/African American	12	17	
Ethnicity–White	154	144	0.602*
MMSE, mean ± SD	24.13 ± 3.21	27.63 ± 2.16	< 0.0001
NfL, pg/mL, mean ± SD	4.72 ± 3.93	3.36 ± 3.04	< 0.0001
GFAP, pg/mL, mean ± SD	155.94 ± 63.35	97.38 ± 46.58	< 0.0001
p‐tau217, pg/mL, mean ± SD	5.05 ± 2.43	2.30 ± 1.18	< 0.0001

*Note*: Statistics represent the Wilcoxon rank‐sum test statistic used to quantify group differences in biomarkers and MMSE scores. Sex differences quantified by chi‐square test. Ethnicity distribution was calculated using Fischer's test due to small group sizes.

Abbreviations: AD, Alzheimer's disease; F, female; GFAP, glial fibrillary acidic protein; M, male; MCI, mild cognitive impairment; MMSE, Mini‐Mental State Examination; NfL, neurofilament light; p‐tau217, phosphorylated tau 217; SD, standard deviation.

### 3.2 Principal components from plasma inflammation markers

**FIGURE 1 alz71642-fig-0001:**
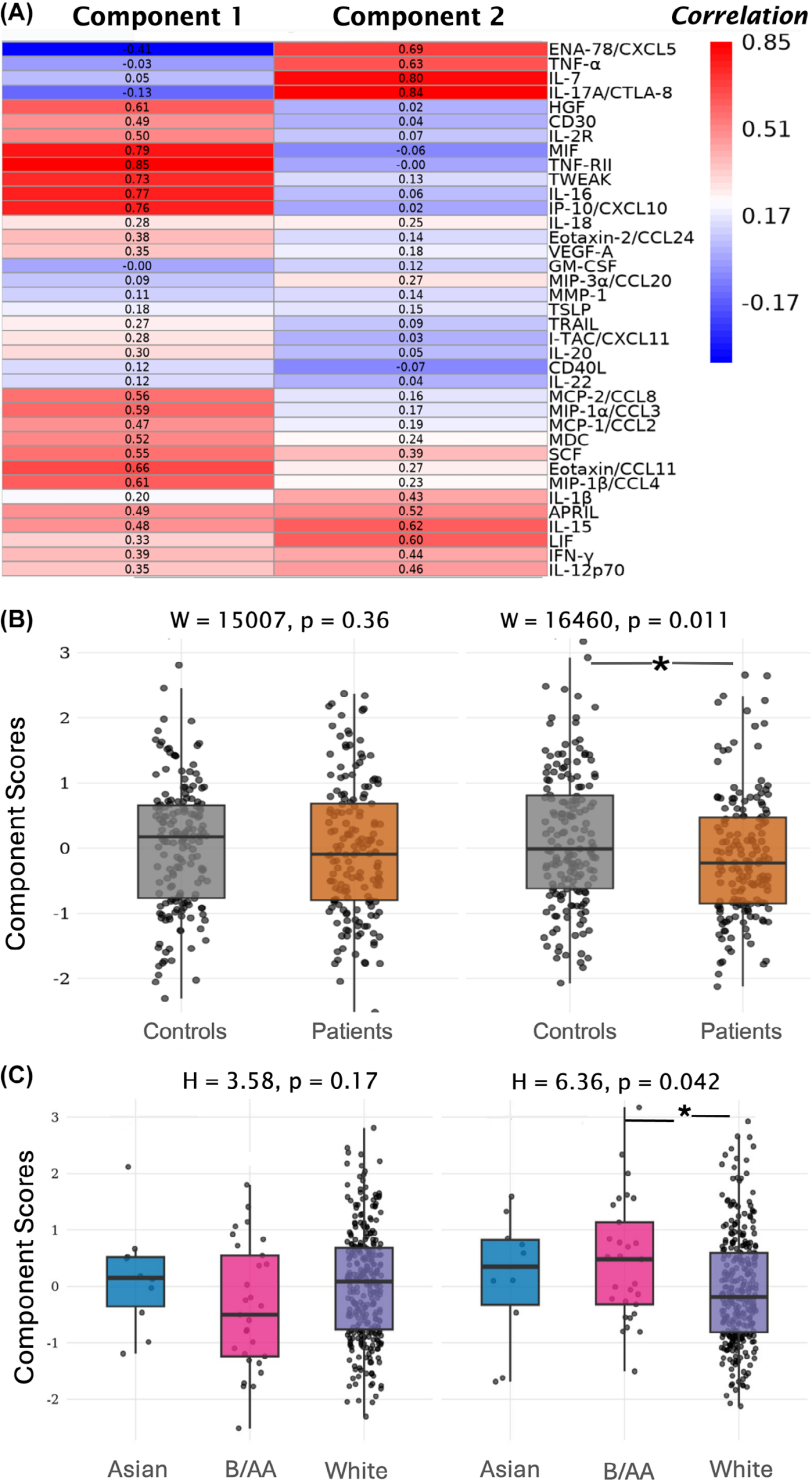
Inflammation profiles and group comparisons. (A) Two resulting significant components from principal component analysis (PCA). The colors indicate the strength of correlations for each cytokine with the components, indicating how strongly these load onto the components. (B) Distribution of Component 1 and Component 2 scores by group. (C) Distribution of component scores by ethnic group.

The PCA identified two significant components across cytokines, with eigenvalues >1 (Component 1: 10.2, Component 2: 3.9) that together cumulatively explained roughly 34% percent of the total data variance (Component 1: 24.2%, Component 2: ≈10%). Component 1 was loaded onto chemokines (CCL11: C‐C motif chemokine ligand 11 or Eotaxin‐1; CXCL10: C‐X‐C motif chemokine ligand 10 or IP‐10), macrophage migration inhibitory factor (MIF) and T helper (Th)1‐associated markers (IL‐16: Interleukin‐16; TNF‐RII), likely reflecting broad innate immune activation and immune cell recruitment. It also included tissue‐remodeling factors such as hepatocyte growth factor (HGH) and TNF‐like weak inducer of apoptosis (TWEAK) (Figure 1A).

On the other hand, Component 2 was mainly represented by pro‐inflammatory cytokines, especially driven by IL‐17A, TNF‐α, IL7, IL15, and interferon‐related signals, indicative of acute cell‐mediated inflammation. Its strong loadings on Th17 and interferon pathways are potentially suggestive of microglial or peripheral innate immune activation, distinct from the Component 1 markers.

Between control and AD groups, Component 1 scores did not differ when Wilcoxon rank‐sum tests were run (Wilcoxon rank‑sum statistic (*W)* = 15007, *p* = 0.36). However, Component 2 scores were significantly higher in the AD group (*W* = 16460, *p* = 0.011) (Figure 1B). Black/African American participants showed higher Component 2 scores than White participants (Kruskal–Wallis chi‑square statistic (*H)* = 6.36, *df* = 2, *p* = 0.042; Dunn post hoc), whereas Component 1 scores did not differ by ethnicity (*H* = 3.58, *df* = 2, *p* = 0.17) (Figure 1C). When including ethnicity in the group comparisons between individuals with AD and controls, the group difference in Component 2 scores remained significant (*β* = 0.29, standard error [SE] = 0.11, Type II analysis of variance [ANOVA] F (1, 333) = 7.12, *p* = 0.008), with an independent effect of ethnicity on Component 2 (F (2, 333) = 3.93, *p* = 0.021). For Component 1, neither ethnicity (F (2, 333) = 2.18, *p* = 0.115) nor groups (F (1, 333) = 0.24, *p* = 0.628) were significant predictors.

### 3.3 Component associations with pathology, neurodegeneration markers, and cognition

**FIGURE 2:**
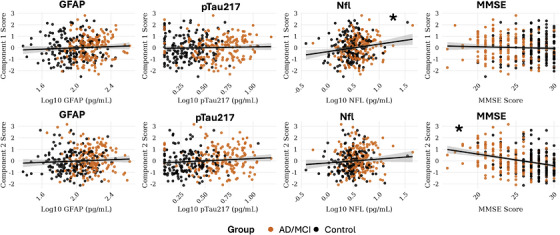
Associations between components, dementia biomarkers, and cognitive scores. Robust regression models were run for each component to test their association with plasma biomarkers and Mini‐Mental State Examination (MMSE) scores in participants with Alzheimer's disease/mild cognitive impairment (AD/MCI) and healthy individuals (Control group) (* indicates significance at *p* < 0.05 threshold).

Neither component was significantly associated with p‐tau217 levels (Component 1: *β* = 0.01, *p* = 0.56; Component 2: *β* = 0.03; *p* = 0.08), indicating that peripherally measured inflammation patterns[Fig alz71642-fig-0001] did not directly reflect AD pathology burden (Figure 2). GFAP plasma levels also did not show significant associations with Component 1 (*β* = 0.02, *p* = 0.17) and Component 2 (*β* = 0.01, *p* = 0.29). Multiple and adjusted R^2^ for the models are reported in Tables S3 and S4.

Regression models with inflammation components and plasma NfL levels identified a positive and significant association between Component 1 and NfL (*β* = 0.03*, p* = 0.006), which remained significant running the model in only patient group only (*β* = 0.03, *p* = 0.03) (Figure S4, Tables S5 and S6). Component 2 did not show significant associations with NfL levels (*β* = 0.02, *p* = 0.11). Pro‐inflammatory cytokine profile Component 2 was significantly and negatively related to cognitive performance (*β* = –1.1, *p* < 0.001), even when the analysis was run in the patient group only (*β* = –1.1, *p* < 0.001) (Figure S3). In contrast, Component 1 scores were not significantly associated with MMSE scores (*β* = –0.2, *p* = 0.27). All associations’ statistical significance were upheld under BH‐FDR multiple comparison corrections (Tables S7 and S8).

### 3.4 Mediation path analysis of inflammation, neurodegeneration, and cognition

**FIGURE 3:**
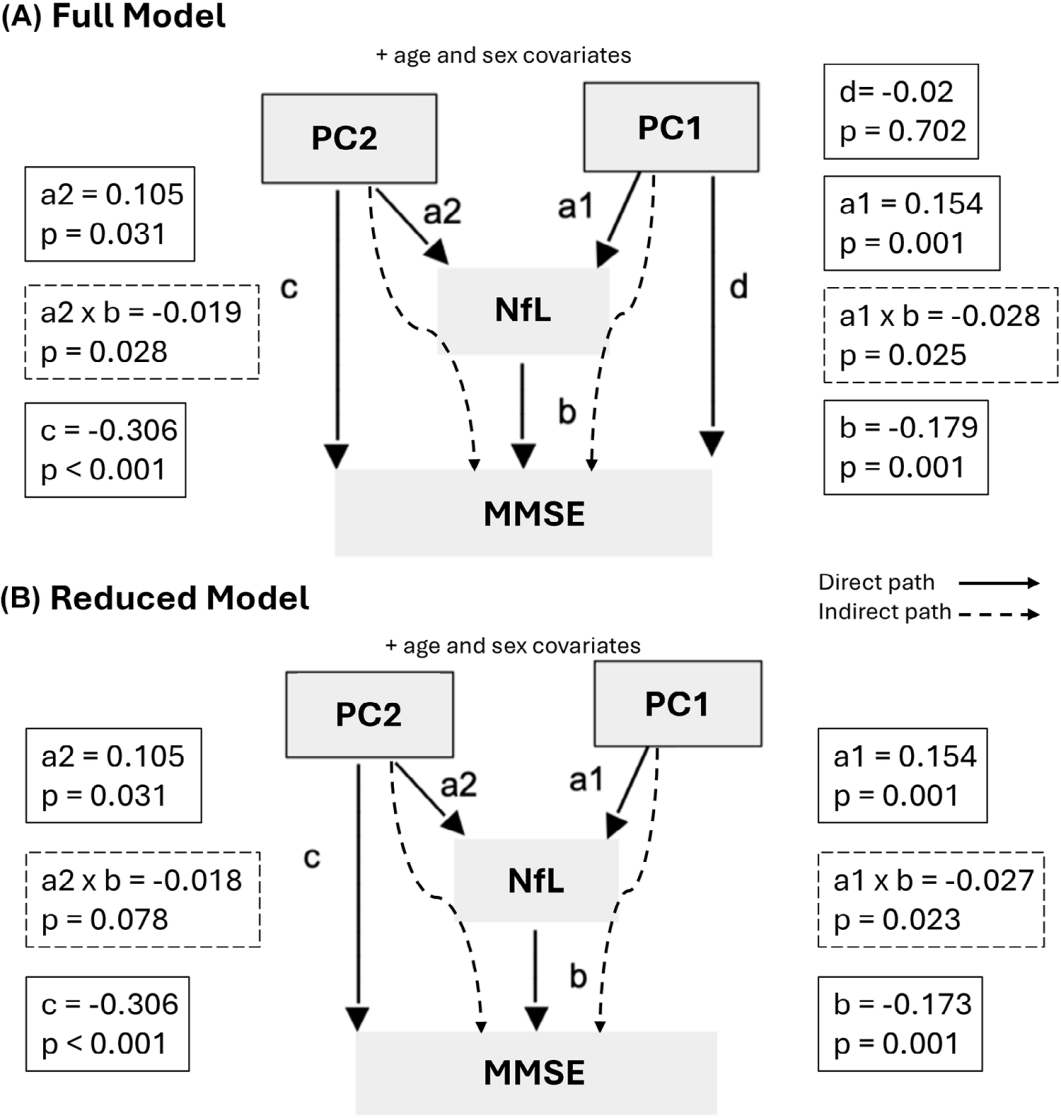
Structural equation modeling of direct and neurofilament light (NfL)–mediated effects of inflammatory components on cognitive scores. (A) Full model including all predictors and pathways fitting with the study hypothesis. (B) The reduced best‐fitting model obtained with the stepwise selection by the Wald test, removing Component 1 ∼ MMSE (Mini‐Mental State Examination) path. Boxes denote observed variables; arrows are retained regression paths.

Given the initial lack of significant associations between cytokine components GFAP and p‐tau217, the latter were not included in the path analysis. The full specified path model (Figure 3A) revealed that Component 2 showed a direct negative association with MMSE (*c* = –0.31, *p* < 0.001), whereas Component 1 showed no direct, significant association with MMSE (*d* = –0.02, *p* = 0.70). NfL was negatively associated with MMSE scores (*b* = –0.179, *p* < 0.001). The associations between Component 1 and NfL (*a1* = 0.154, *p* = 0.001) and between Component 2 and NfL (*a2* = 0.105, *p* = 0.03) were statistically significant, making both indirect effects significant (Figure 3). All path coefficients reported were standardized. AIC for this model was 4583.2.

Following inspection of the full‑path model (Table S9), we tested a reduced model removing the direct path of Component 1 to MMSE (Path d) using a Wald test. Results did not exceed the significance threshold (*p* = 0.95), so we removed the path and refit the reduced model. Global fit indices indicated an uninformatively perfect fit (RMSEA = 0.00, Comparative Fit Index (CFI) = 1.00, Tucker‐Lewis Index (TLI) = 1.08), which is likely driven by low degrees of freedom in almost saturated models. However, the AIC of 4577.6 indicates this model is more parsimonious than the full one. This reduced best‐fitting model included the same direct negative effect of Component 2 on MMSE (*c* = –0.31, *p* < 0.001) (Figure 3B). Component 1 still positively predicted NfL levels (*a1* = 0.154, *p* = 0.001), and NfL negatively predicted MMSE scores (*b1* = –0.173, *p* = 0.001). The NfL‐mediated pathway from Component 1 to MMSE was small, but significant (*a1·b* ≈ –0.03, *p* = 0.023) and the mediation effect was tested with bootstrapping (4000 draws; 95% BCa CI [–0.184, –0.026]) In this reduced model the indirect effect of Component 2 (a2*·b*) was non‐significant. (*p* = 0.078) Therefore, the total effects of Components 1 (indirect path only) and 2 (direct path only) were significant on MMSE (Table S10).

## 4 DISCUSSION

By analyzing a larger and more ethnically diverse cohort, this study identified clinically relevant inflammatory cytokine signatures differentially associated with neurodegeneration and clinical outcomes in individuals with AD.

Previous peripheral cytokine studies highlighted differential patterns between individuals on the AD spectrum and healthy volunteers, including increased levels of plasma and serum TNF‐α, and IL‐6.^35^, ^36^, ^37^, ^38^ However, most studies have been conducted in small, single‐center cohorts, with limited population diversity, focusing on investigating single markers. Here, we applied data‐driven approaches to investigate cytokine co‐expression variation and identify blood‐based inflammation signatures.

Component 1 loaded onto pro‐inflammatory markers, with highest loadings by TNF‐RII, MIF, IL‐16, and CXCL10. Previous research found MIF elevated in both MCI and AD.^39^ An AD CSF study ranked MIF among the top contributors of a principal inflammatory component.^40^ Moreover, TNF‐RII, the highest‐loading marker in this analysis, has been explored as a putative neuroprotective therapeutic target.^41^, ^42^ Higher levels of MIF and TNF‐RII are suggestive of an inflammatory‐remodeling axis where damage‐associated and repair‐associated signals increase in tandem.^43^

Component 2 scores differed between controls and individuals with AD. This component loaded mainly onto pro‐inflammatory cytokines (TNF‐α, IL‐7, IL‐17A, and CXCL5). Plasma and CSF studies have reported elevated TNF‐α in individuals with AD, with higher levels associated with greater disease severity.^44^, ^45^ The positive loading of IL‐17A on this component is notable, given its canonical association with Th17 responses.^46^, ^47^ However, the high loadings of IL‐7 and IL‐15 suggest that this component may reflect broader lymphocyte‐associated responses rather than a single effector axis^44^, ^46^ The co‐variation captured here is therefore unlikely to represent a single pathway; rather, it reflects shared downstream signaling among cytokines whose origins span monocytes, T cells, and non‐immune tissue sources.^48^ These patterns should be interpreted cautiously considering that most of the markers analyzed here capture changes spanning innate and adaptive immunity.

Although the current cohort included smaller numbers of participants from non‐White groups, the difference between ethnicities is of interest and highlights the need for more research in diverse cohorts. Ethnic variation in immune function arises from a complex interplay of genetic, environmental, and sociocultural factors.^18^ Polymorphisms in immune‐regulatory genes, differential exposure to pathogens and environmental stressors, different chronic illness burdens, and inequities in structural determinants of health all contribute to baseline inflammatory tone and the balance of specific cytokine pathways.^49^, ^50^, ^51^, ^52^

Using our PCs in a pre‑specified path mediation analysis, we identified two pathways linking inflammation to cognitive impairment. The model indicated a direct pathway from Component 2 to lower cognitive scores and an indirect pathway where neuroaxonal damage (NfL), mediated the effect of Component 1 on cognitive decline. These results, align with and build upon previous findings linking high peripheral inflammation with faster clinical progression in participants with AD, where higher levels of blood‐based TNF‐α and various interlukins correlated with pathology burden and clinical decline.^53^

Although chronic low‐grade inflammation may not directly impair cognition, acute pro‐inflammatory responses characterized by specific signaling cascades and T‐helper cell recruitment can provoke delirium‐like symptoms or tau‐independent synaptic dysfunction.^54^ Clinically, individuals with AD and inflammatory comorbidities often exhibit acute delirium‐related confusion and more rapid deterioration.^54^ Thus, elevated central inflammation measured peripherally may mirror some concurrent neuroinflammatory processes, indexed by inflammation‐targeting brain PET, that disrupt cognitive networks.^55^, ^56^ Our findings also suggest that chronic and low‐grade inflammation may be able to gradually compromise neural integrity, which in turn affects cognitive performance. Previous studies reported that blood‐derived NfL levels correlate with pro‐inflammatory blood‐based markers, including IL‐1β, IL‐6 and TNF‐α signaling via soluble TNF receptors, underscoring the link between peripherally captured inflammation and neuronal injury.^57^, ^58^ TNF‐driven inflammation has been associated with necroptosis in post‐mortem histochemical analyses in AD, supporting this notion of a link between immune activation and neurodegeneration.^59^

The differential effects of the two components on cognition suggest that not all inflammation profiles are directly or equally detrimental to cognition, rather the elevation of specific cytokine pathways may be linked with cognitive deficits. Component 1 likely indexes an early, compensatory innate response to general neurodegeneration, explaining its comparable levels across AD/MCI and control groups. Therefore, these generalized inflammation patterns, indexed by Component 1, might contribute to cumulative neuronal damage, potentially through vascular inflammation or sustained cytokine exposure which impair brain homeostasis and neuroprotective processes.^60^, ^61^ The release of chemokines, including MIF and CXCL10, could help recruit peripheral immune cells, forming a self‐sustaining neuroinflammatory loop. Together, dual modes of immune activation illustrate how inflammation can fuel neurodegeneration and disrupt cognitive processes, with outcomes determined by context and activation profile.^62^

Both inflammatory components did not significantly associate with plasma GFAP levels. This may be explained by the fact that these components captured inflammatory signatures that do not directly index astrocyte reactivity. Consistent with this, a previous study found weak correlations between plasma GFAP and individual inflammatory cytokine levels, like TNF‐α and various interleukins.^63^ Similarly, the absence of a statistically significant association between plasma cytokines and p‑tau217 levels in this cohort is suggestive of partial independence between inflammation and amyloid progression. Preclinical and imaging studies nonetheless indicate bidirectional interactions between Aβ pathology, microglial activation, and tau phosphorylation.^64^, ^65^ Longitudinal multi‐tracer PET studies in MCI/AD suggest that microglial activation can co‐localize with tau pathology and, in some regions, may precede subsequent tau accumulation.^4^ However, these regional interactions captured with PET may not translate directly to peripheral measurements. This dissociation underscores the complex dynamics of inflammatory processes across disease progression with differential involvement of central and peripheral immune responses at different stages.^66^, ^67^ In addition, a proportion of the cytokine variance was still not explained by both components, which may be why statistically significant associations with GFAP and p‐tau217 plasma levels were absent.

This study has several limitations. The PCA approach captures statistical covariance, and therefore mechanistic interpretations about biological pathways need to be addressed with caution. The study's cross‐sectional design precludes causal inferences, offering no information as to whether elevated inflammatory signatures precede or follow neuronal injury, and time since diagnosis was unavailable in the dataset, which limits our ability to account for inflammation heterogeneity linked to disease duration. Longitudinal studies integrating imaging endpoints and interventional designs will be essential to determine how inflammatory profiles influence disease trajectory. Although this study benefitted from a relatively large sample size, bigger cohorts may be needed to detect smaller effects sizes, particularly when considering NfL's modest association with Component 2 (Figure 2). Although more sensitive approaches to quantify cytokines in plasma are now available to the scientific and research community (e.g., NUcleic acid‐linked Immuno‐Sandwich Assay (NULISA)) and have recently been leveraged to yield insights on inflammation across neurodegenerative disorders, the 65‐plex Luminex assay was the only approach available in the BioHermes dataset.^68^ Our findings will need to be validated by future studies with more sensitive assays detecting larger numbers of inflammatory markers. In addition, peripheral cytokine levels are susceptible to comorbidities, lifestyle factors, and polypharmacy, and future research should explore how these factors influence peripheral inflammation.^69^ Without access to TSPO‐PET and/or CSF measures, the association of peripheral inflammatory measures with direct measures of brain inflammation could not be assessed.^11^

It is important also to consider that participants involved in medical research studies exhibit healthier profiles and higher education levels than the average population, and our smaller Black/African American and Asian subgroups limited power for in‐depth subgroup analyses. When considering ethnicity differences, it is important that future research in larger cohorts aims to disentangle immune changes related to genetic differences from those that could be secondary to other health conditions, environmental factors, or social context, and contribute to disease burden. To validate current findings, future studies in dementia need to engage with and recruit more ethnically diverse participants (e.g., Health & Aging Brain Study – Health Disparities (HABS‐HD).^70^

Post‐mortem studies indicate that co‐pathologies are common in patients with AD with α‐synuclein, Transactive Response DNA binding Protein 43 kDa (TDP‐43), and vascular co‐pathologies present in a substantial proportion of clinically diagnosed cases.^71^ However, neuropathological confirmation at autopsy was not available and co‐pathologies were not directly assessed. We therefore cannot exclude that co‐pathologies contributed to the inflammatory variance observed. Future work incorporating α‐synuclein and TDP‐43 assays, will be important to determine whether inflammatory profiles are modulated by the presence or absence of other brain pathologies, in addition to AD.

This work identified two distinct blood‐derived inflammatory profiles in AD that differ in their associations with pathology and cognition. Although trials targeting inflammation have shown mixed results, data‐driven characterization of blood‐based inflammation signatures can help clarify the heterogeneity of AD progression and monitor responses in immuno‐targeting trials. Our results align with emerging evidence that effective drug development may require tackling both neurodegenerative pathology and immune dysregulation, and combination immunomodulatory strategies that dampen harmful cytokine cascades while preserving protective innate responses have been proposed as next‐generation therapies.^72^ Combined with neurodegeneration markers such as NfL, scalable inflammatory panels could aid patient stratification and enrich trial participants by inflammation profile.

## CONFLICT OF INTEREST STATEMENT

The authors have no conflicts of interest to report related to this work. Unrelated to this work, J.T.O. has received honoraria for work as Data and Safety Monitoring Board (DSMB chair or member for TauRx, Axon, Eisai, and Novo Nordisk; has acted as a consultant for Biogen and Roche; and has received research support from Alliance Medical and Merck. M.M. has consulted for Astex Pharmaceuticals. W.A.M. has received research funding from Takeda Pharmaceuticals and is a founder and consultant for Trimtech Therapeutics. A.H.H. has received honoraria from Eli‐Lilly and from the National Institute on Aging (NIA). He serves as a consultant for AriBio Co. Ltd and chairs the DementiasPlatform UK Vascular Experimental Medicine group. A.H.H.’s group has received research funds from the UK Medical Research Council (MR/R005567/1, MR/T033371/1), British Heart Foundation (PG/20/10397, SP/F/22/150042, PG/24/11757), UK Alzheimer's Society (AS‐DTC‐24‐004), and Alzheimer's Drug Discovery Foundation (20140901). Author disclosures are available in the Supporting Information.

## CONSENT STATEMENT

All participants provided written informed consent prior to undertaking any study procedures. The study was conducted in accordance with the ethical standards of the 1964 Declaration of Helsinki and its later amendments, and was approved by Advarra, a central institutional review board (Reference Number Pro00046018), with approval from the appropriate institutional review boards at each participating site.

## Supporting information




**Supporting Information**: alz71641‐Sup‐0002‐SuppMat.docx

**Supporting Information**: alz71641‐Sup‐0001‐ICMJE.pdf
