# Supplementary material for: Inflammation profiles in Alzheimer's disease relate to cognition and neurodegeneration
Source: Alzheimers Dement. 2026 Jun 27;22(7):e71642. doi: 10.1002/alz.71642 (PMC13309851; doi:10.1002/alz.71642)
Supplement: Supplementary file 1 — Supporting Information: alz71641‐Sup‐0002‐SuppMat.docx [file ALZ-22-e71642-s001.docx]

**Supplementary Materials**

**Table 1.** Summary of cytokines (values in pg/ml) following MICE imputation, split by Control and AD/MCI with mean, standard deviation and median shown per group.

| **Inflammatory marker** | **AD/MCI (n)** | **AD/MCI**  **Mean ± SD** | **AD/MCI Median** | **Control (n)** | **Control Mean ± SD** | **Control Median** |
| --- | --- | --- | --- | --- | --- | --- |
| APRIL | 171 | 965.24 ± 1030.70 | 813.55 | 166 | 895.40 ± 1041.76 | 638.94 |
| CD30 | 171 | 509.15 ± 345.81 | 411.19 | 166 | 446.01 ± 237.69 | 392.94 |
| CD40L | 171 | 176.96 ± 958.80 | 35.16 | 166 | 100.31 ± 171.84 | 33.56 |
| ENA-78 / CXCL5 | 171 | 306.85 ± 309.50 | 214.67 | 166 | 274.76 ± 282.99 | 182.80 |
| Eotaxin / CCL11 | 171 | 5.39 ± 3.22 | 4.36 | 166 | 5.43 ± 5.21 | 4.38 |
| Eotaxin-2 / CCL24 | 171 | 616.44 ± 1481.51 | 333.66 | 166 | 577.55 ± 963.06 | 357.38 |
| GM-CSF | 171 | 4.90 ± 6.19 | 3.51 | 166 | 4.53 ± 2.66 | 3.70 |
| HGF | 171 | 66.98 ± 34.19 | 61.98 | 166 | 69.13 ± 51.76 | 58.75 |
| I-TAC / CXCL11 | 171 | 14.02 ± 22.20 | 8.79 | 166 | 14.29 ± 25.57 | 7.51 |
| IFN-γ | 171 | 9.45 ± 7.09 | 8.33 | 166 | 8.42 ± 4.31 | 7.81 |
| IL-1β | 171 | 4.22 ± 2.17 | 3.80 | 166 | 3.74 ± 2.65 | 3.00 |
| IL-12p70 | 171 | 0.85 ± 0.44 | 0.80 | 166 | 0.83 ± 0.44 | 0.75 |
| IL-15 | 171 | 2.35 ± 1.22 | 2.22 | 166 | 2.15 ± 1.10 | 1.97 |
| IL-16 | 171 | 48.14 ± 28.71 | 44.20 | 166 | 47.01 ± 42.10 | 36.46 |
| IL-17A | 171 | 11.31 ± 9.92 | 8.82 | 166 | 9.13 ± 8.46 | 6.58 |
| IL-18 | 171 | 49.12 ± 27.69 | 41.96 | 166 | 47.64 ± 28.09 | 44.02 |
| IL-20 | 171 | 11.74 ± 29.91 | 3.21 | 166 | 9.83 ± 15.16 | 3.71 |
| IL-22 | 171 | 168.07 ± 737.49 | 29.88 | 166 | 149.30 ± 452.68 | 32.42 |
| IL-2R | 171 | 2411.85 ± 1734.62 | 2031.80 | 166 | 2047.87 ± 1604.19 | 1760.03 |
| IL-7 | 171 | 0.63 ± 0.38 | 0.60 | 166 | 0.53 ± 0.38 | 0.46 |
| IP-10 / CXCL10 | 171 | 9.64 ± 6.29 | 8.18 | 166 | 9.84 ± 8.11 | 7.76 |
| LIF | 171 | 1.74 ± 0.94 | 1.63 | 166 | 1.53 ± 0.89 | 1.44 |
| MCP-1 / CCL2 | 171 | 32.46 ± 19.81 | 29.11 | 166 | 30.89 ± 17.53 | 29.20 |
| MCP-2 / CCL8 | 171 | 6.35 ± 3.28 | 6.07 | 166 | 6.43 ± 3.08 | 5.78 |
| MDC | 171 | 224.86 ± 174.87 | 188.00 | 166 | 205.78 ± 183.74 | 146.73 |
| MIF | 171 | 11.00 ± 5.21 | 10.33 | 166 | 10.59 ± 5.76 | 9.28 |
| MIP-1α / CCL3 | 171 | 6.27 ± 8.59 | 3.97 | 166 | 6.54 ± 7.64 | 3.76 |
| MIP-1β / CCL4 | 171 | 5.98 ± 5.05 | 4.54 | 166 | 5.96 ± 6.21 | 4.26 |
| MIP-3α / CCL20 | 171 | 11.28 ± 13.21 | 8.64 | 166 | 10.33 ± 7.24 | 8.68 |
| MMP-1 | 171 | 8.85 ± 10.86 | 5.45 | 166 | 6.83 ± 7.69 | 4.39 |
| SCF | 171 | 5.34 ± 3.73 | 4.52 | 166 | 5.01 ± 4.08 | 4.00 |
| TNF-RII | 171 | 93.90 ± 53.61 | 86.27 | 166 | 94.66 ± 70.60 | 76.26 |
| TNF-α | 171 | 3.39 ± 5.22 | 2.42 | 166 | 2.73 ± 2.75 | 2.29 |
| TRAIL | 171 | 50.36 ± 200.05 | 14.98 | 166 | 41.57 ± 130.80 | 11.86 |
| TSLP | 171 | 4.18 ± 5.28 | 3.03 | 166 | 7.35 ± 15.81 | 3.25 |
| TWEAK | 171 | 542.47 ± 254.43 | 500.23 | 166 | 515.86 ± 285.43 | 444.71 |
| VEGF-A | 171 | 121.99 ± 332.57 | 53.53 | 166 | 153.94 ± 422.44 | 54.61 |

Fi**g 1.** Principal component analysis result excluding cytokines with >50% missing data. Pattern of cytokine variation is similar to analysis run on data with >75% missingness threshold.


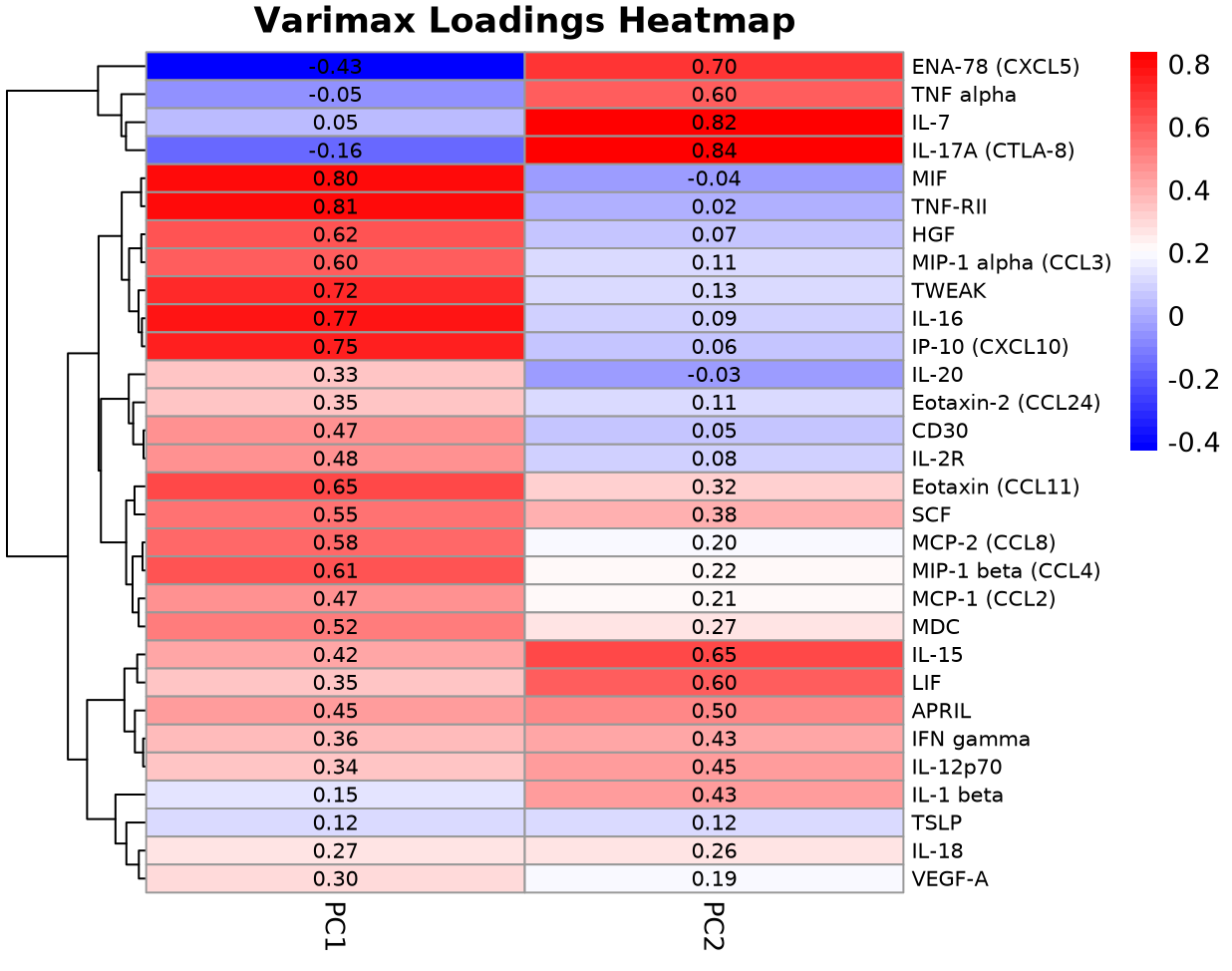


**Fig 2.** Plot of the first two components, highlighting the 12 outliers identified by the Mahalanobis Outlier Analysis which were excluded from further analyses*.*


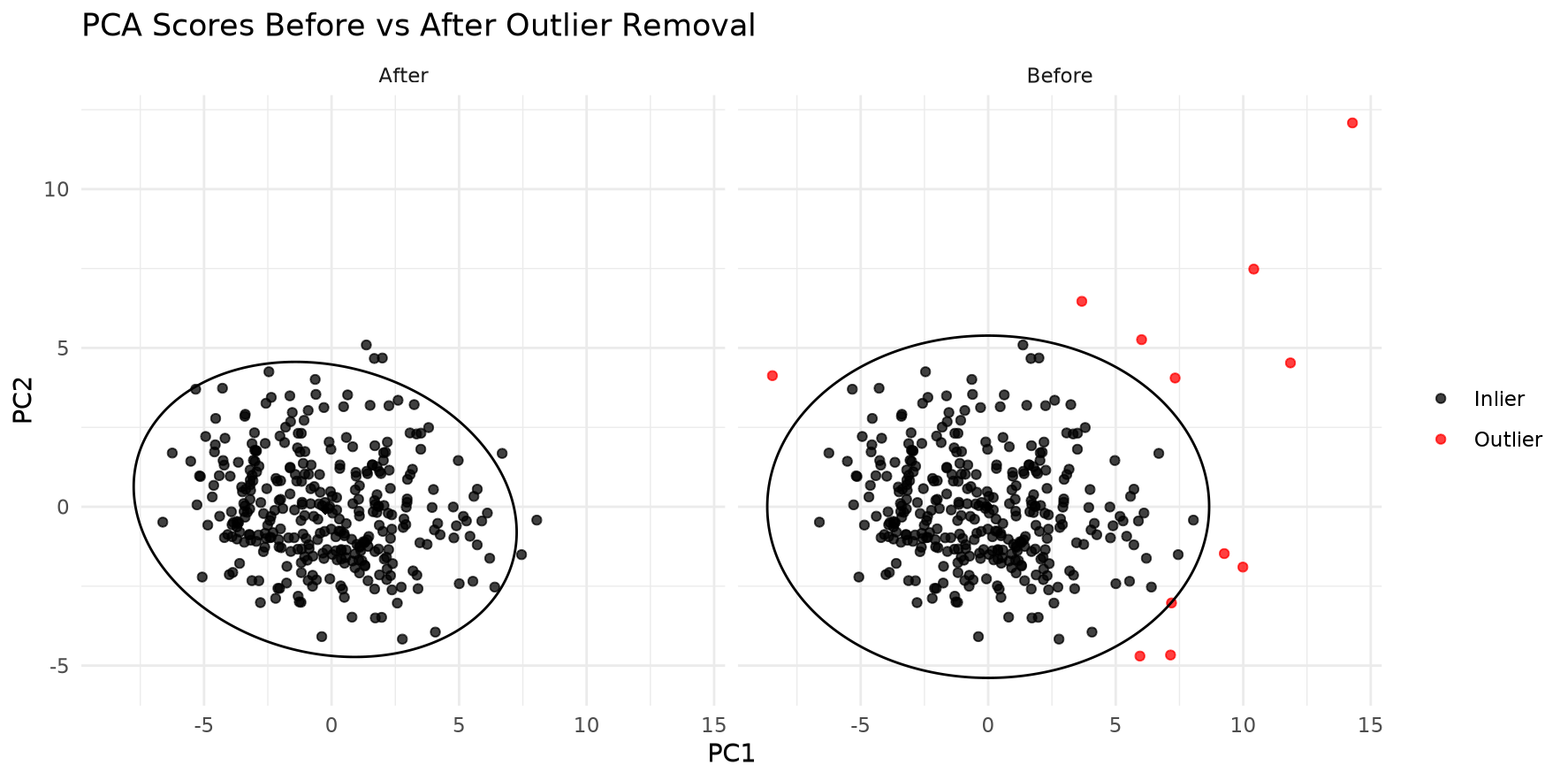


**Fig 3.** Distribution of within-group prevalences of different medical conditions obtained from medical history data. We reported conditions with prevalence > 0.05 in the cohort.


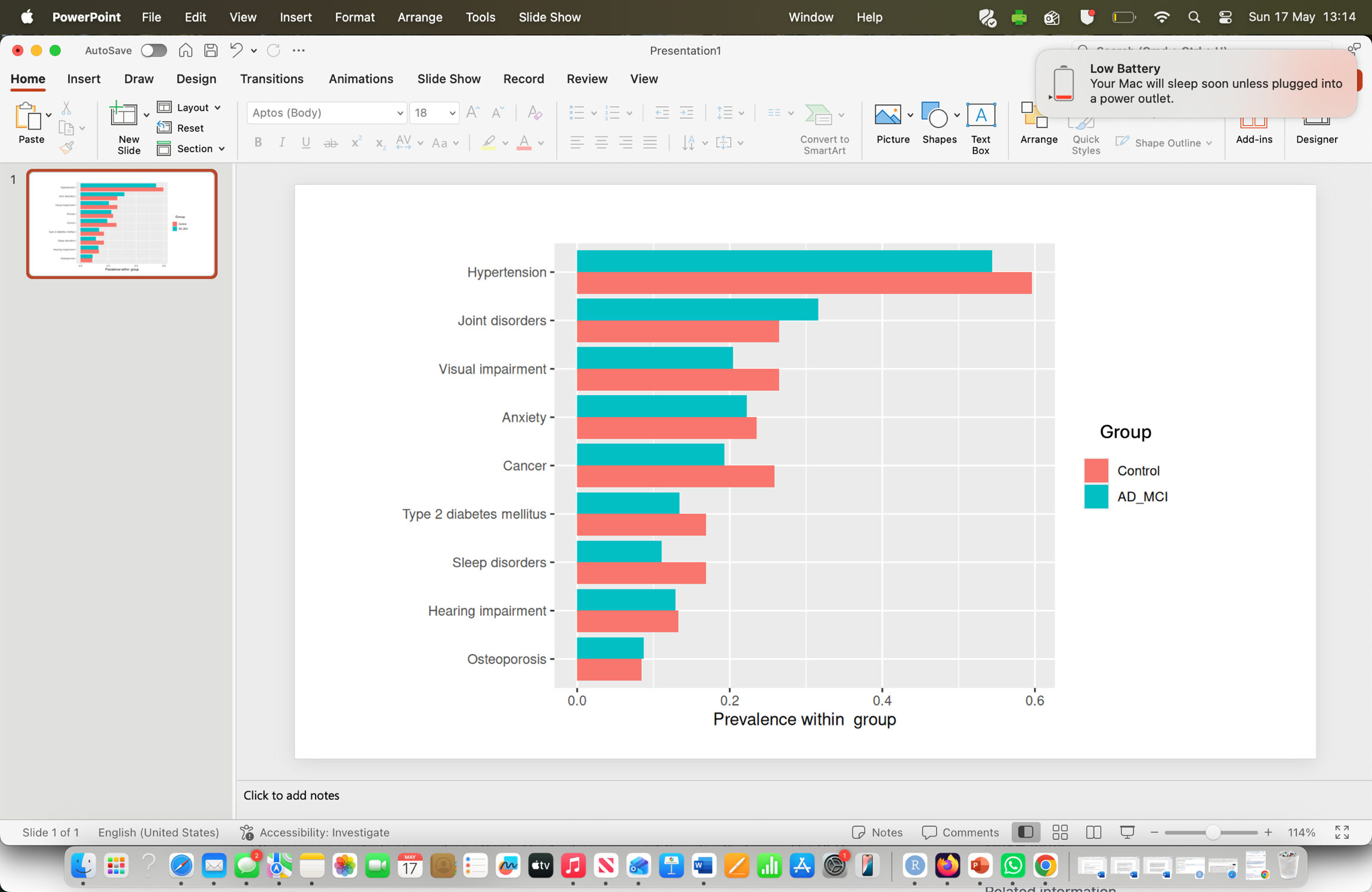
**Fig 4.** Robust regression results including only patients (no controls) in the analyses.


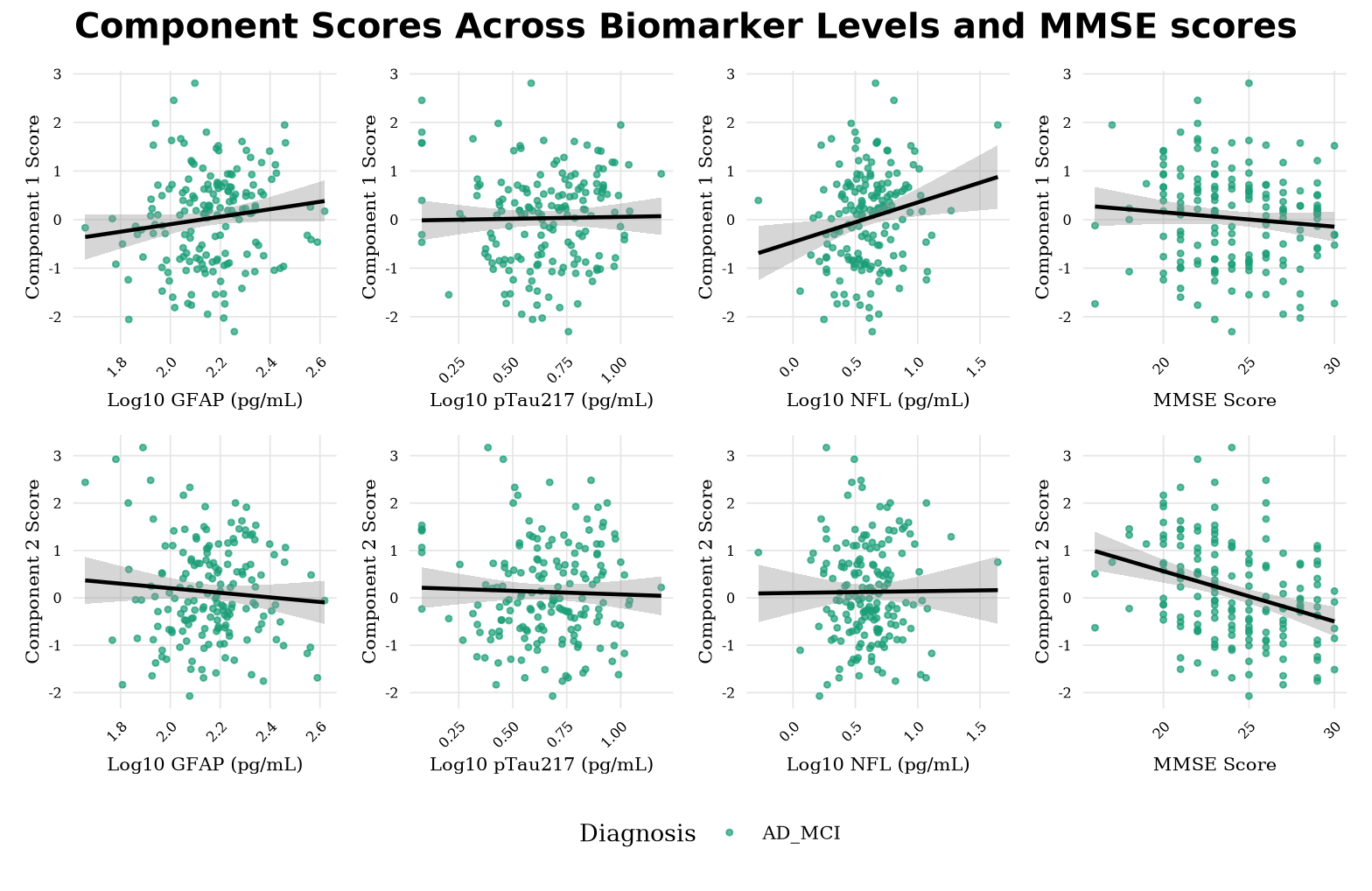


**Table 2**. Summary of demographics and biomarker results by ethnic groups prior to outlier removal from PCA.

| **Self-defined Ethnicity** | **Group** | **N** | **Female (%)** | **Male (%)** | **Age Mean** | **Age SD** | **MMSE (mean ± SD)** | **NFL (mean ± SD)** | **GFAP (mean ± SD)** | **pTau217 (mean ± SD)** |
| --- | --- | --- | --- | --- | --- | --- | --- | --- | --- | --- |
| Asian | AD/MCI+ | 5 | 1 (20.0%) | 4 (80.0%) | 75.0 | 3.9 | 21.80 ± 2.49 | 7.53 ± 3.94 | 207.68 ± 107.28 | 6.20 ± 2.84 |
| Asian | Control | 5 | 3 (60.0%) | 2 (40.0%) | 74.4 | 7.0 | 26.80 ± 2.95 | 3.24 ± 1.12 | 88.15 ± 35.70 | 2.18 ± 0.67 |
| Black/African American | AD/MCI+ | 12 | 9 (75.0%) | 3 (25.0%) | 72.2 | 5.4 | 22.58 ± 2.97 | 4.50 ± 3.08 | 136.50 ± 50.90 | 4.25 ± 2.15 |
| Black/ African American | Control | 17 | 9 (52.9%) | 8 (47.1%) | 74.4 | 5.5 | 25.47 ± 2.98 | 2.76 ± 1.17 | 87.55 ± 33.51 | 2.07 ± 0.74 |
| White | AD/MCI+ | 154 | 73 (47.4%) | 81 (52.6%) | 75.2 | 5.9 | 24.33 ± 3.20 | 4.64 ± 3.97 | 155.78 ± 62.08 | 5.07 ± 2.43 |
| White | Control | 144 | 67 (46.5%) | 77 (53.5%) | 74.1 | 6.0 | 27.91 ± 1.87 | 3.43 ± 3.23 | 98.86 ± 48.23 | 2.33 ± 1.23 |

**Table 3.** All coefficients obtained from the robust regression models run on both patients and healthy participants (SE – standard error; significance codes: p < 0.001**,**  p < 0.01, p < 0.05, p < 0.1).

| **Outcome** | **Predictor** | **β (Estimate)** | **SE** | **p-value** |
| --- | --- | --- | --- | --- |
| **GFAP** | PC1 | 0.0152 | 0.0110 | 0.167 |
|  | PC2 | 0.0124 | 0.0117 | 0.292 |
|  | Age | 0.0117 | 0.0018 | < 0.001 *** |
|  | Sex | –0.1051 | 0.0220 | < 0.001 *** |
| **NFL** | PC1 | 0.0306 | 0.0110 | 0.0059 ** |
|  | PC2 | 0.0182 | 0.0115 | 0.1138 |
|  | Age | 0.0140 | 0.0020 | < 0.001 *** |
|  | Sex | –0.0448 | 0.0225 | 0.0476 * |
| **MMSE** | PC1 | –0.2012 | 0.1825 | 0.271 |
|  | PC2 | –1.1025 | 0.1749 | < 0.001 *** |
|  | Age | –0.0337 | 0.0312 | 0.281 |
|  | Sex | 0.0280 | 0.3522 | 0.937 |
| **pTau217** | PC1 | 0.0099 | 0.0169 | 0.5595 |
|  | PC2 | 0.0290 | 0.0163 | 0.0759 . |
|  | Age | 0.0040 | 0.0025 | 0.1159 |
|  | Sex | 0.0253 | 0.0305 | 0.4069 |

**Table 4.** Multiple and Adjusted R-squared values for each predictor in robust regression models run on both patient and control groups.

| **Outcome** | **Multiple R²** | **Adjusted R²** |
| --- | --- | --- |
| **GFAP** | 0.177 | 0.167 |
| **NFL** | 0.179 | 0.169 |
| **MMSE** | 0.119 | 0.109 |
| **pTau217** | 0.023 | 0.011 |

**Table. 5** All coefficients obtained from the robust regression models run on AD/MCI individuals only (no controls)

| **Outcome** | **Predictor** | **Estimate** | **SE** | **p-value** |
| --- | --- | --- | --- | --- |
| **GFAP** | PC1 | 0.01817 | 0.01321 | 0.1708 |
|  | PC2 | −0.00793 | 0.01466 | 0.5891 |
|  | Age | 0.00755 | 0.00219 | 0.000707 *** |
|  | Sex | −0.08153 | 0.02392 | 0.000821 *** |
| **NFL** | PC1 | 0.03377 | 0.01572 | 0.0332 * |
|  | PC2 | 0.00275 | 0.01588 | 0.8628 |
|  | Age | 0.01033 | 0.00244 | < 0.001 *** |
|  | Sex | −0.06063 | 0.03062 | 0.0494 * |
| **pTau217** | PC1 | 0.01848 | 0.02014 | 0.360 |
|  | PC2 | 0.00490 | 0.01738 | 0.779 |
|  | Age | –0.00150 | 0.00252 | 0.552 |
|  | Sex | –0.02933 | 0.03434 | 0.394 |
| **MMSE** | PC1 | −0.44124 | 0.22986 | 0.0566 . |
|  | PC2 | −1.08469 | 0.21838 | 0.00000168 *** |
|  | Age | 0.03787 | 0.04808 | 0.4320 |
|  | Sex | 0.07907 | 0.49293 | 0.8728 |

**Table 6.** Multiple and Adjusted R-squared values for robust regression predictors run in AD/MCI patients only.

| **Outcome** | **Multiple R²** | **Adjusted R²** |
| --- | --- | --- |
| **GFAP** | 0.152 | 0.131 |
| **NFL** | 0.124 | 0.103 |
| **pTau217** | 0.012 | -0.011 |
| **MMSE** | 0.134 | 0.113 |

**Table 7.** The BH-FDR corrected estimates for Component 1 and Component 2 run across plasma biomarker linear models. Estimates (Est), standard errors (SE), test statistics (z_t), raw p-values (p), and Benjamini–Hochberg FDR–adjusted p-values (q_BH) for Component 1, Component 2, age, and sex.

| **outcome** | **predictor** | **estimate** | **SE** | **statistic** | **p** | **BH-FDR corrected q-value** |
| --- | --- | --- | --- | --- | --- | --- |
| NFL | PC1 | 0.0306 | 0.0110 | 2.77 | 0.00590 | 0.0177 |
| GFAP | PC1 | 0.0152 | 0.0110 | 1.38 | 0.167 | 0.251 |
| pTau217 | PC1 | 0.00986 | 0.0169 | 0.584 | 0.560 | 0.560 |
| pTau217 | PC2 | 0.0290 | 0.0163 | 1.78 | 0.0759 | 0.171 |
| NFL | PC2 | 0.0182 | 0.0115 | 1.59 | 0.114 | 0.171 |
| GFAP | PC2 | 0.0124 | 0.0117 | 1.06 | 0.292 | 0.292 |

**Table 8**. FDR was controlled within MMSE predictors. Estimates (Est), standard errors (SE), test statistics (z), raw p-values, and Benjamini–Hochberg FDR–adjusted p-values (q_BH) for both components, age, and sex.

| **term** | **estimate** | **SE** | **statistic** | **p** | **BH-FDR corrected**  **q-value** |
| --- | --- | --- | --- | --- | --- |
| PC2 | -1.10248 | 0.17486 | -6.3050 | <0.001 | <0.001 |
| PC1 | -0.20123 | 0.18254 | -1.1024 | 0.271 | 0.375 |
| Age | -0.03370 | 0.03121 | -1.0797 | 0.281 | 0.375 |
| Sex | 0.02797 | 0.35223 | 0.0794 | 0.937 | 0.937 |

**Table 9.**  Full-path SEM-style mediation analysis of cytokine principal components, NfL, and MMSE. Results from testing whether principal components derived from the cytokine principal components relate to MMSE directly and indirectly via plasma NfL, adjusting for age and sex. Cells report unstandardized coefficients (Estimate), standard errors (Std. Error), z-values, and p-values; standardized effects are provided as Std. (all) Both principal components were not allowed to co-vary due to the orthogonality of their relationship.

| **Path / Parameter** | **Estimate** | **Std. Error** | **z-value** | **p-value** | **Std. (all)** |
| --- | --- | --- | --- | --- | --- |
| **Regressions** |  |  |  |  |  |
| MMSE ~ PC1 **(d)** | -0.065 | 0.170 | -0.383 | 0.702 | -0.020 |
| MMSE ~ PC2 **(c)** | -0.994 | 0.161 | -6.160 | <0.001 | -0.306 |
| MMSE ~ NfL **(b1)** | -0.008 | 0.002 | -3.302 | 0.001 | -0.179 |
| MMSE ~ age | 0.013 | 0.031 | 0.412 | 0.680 | 0.023 |
| MMSE ~ sex(male) | -0.013 | 0.328 | -0.041 | 0.967 | -0.002 |
| NfL ~ PC1 **(a1)** | 10.876 | 3.420 | 3.180 | 0.001 | 0.154 |
| NfL ~ PC2 **(a2)** | 7.408 | 3.429 | 2.160 | 0.031 | 0.105 |
| NfL ~ age | 4.173 | 0.589 | 7.081 | <0.001 | 0.350 |
| NfL ~ sex(male) | -16.008 | 6.955 | -2.302 | 0.021 | -0.114 |
|  |  |  |  |  |  |
| **Defined (Indirect / Total Effects)** |  |  |  |  |  |
| Indirect effect PC1  (a1 × b1) | -0.090 | 0.040 | -2.243 | 0.025 | -0.028 |
| Indirect effect PC2  (a2 × b1) | -0.061 | 0.035 | -1.737 | 0.082 | -0.019 |
| Total effect PC1 | -0.155 | 0.173 | -0.896 | 0.370 | -0.048 |
| Total effect PC2 | -1.055 | 0.161 | -6.555 | 0.000 | -0.325 |

**Table 10.** Wald test trimmed SEM-style path analysis of cytokine principal components, NfL, and MMSE. Results from testing better fitting SEM-style path analysis with MMSE ~ Component 1 path removed. Cells report unstandardized coefficients (Estimate), standard errors (Std. Error), z-values, and p-values; standardized effects are provided as and Std. (all)

| **Path / Parameter** | **Estimate** | **Std. Error** | **z-value** | **p-value** | **Std. (all)** |
| --- | --- | --- | --- | --- | --- |
| **Regressions** |  |  |  |  |  |
| MMSE ~ PC2 (c) | -0.994 | 0.160 | -6.209 | <0.001 | -0.306 |
| MMSE ~ NfL (*b*) | -0.008 | 0.002 | -3.427 | 0.001 | -0.173 |
| NfL ~ PC1 (*a1*) | 10.876 | 3.420 | 3.180 | 0.001 | 0.154 |
| NfL~ PC2 (*a2*) | 7.408 | 3.429 | 2.160 | 0.031 | 0.105 |
| NfL ~ age | 4.173 | 0.589 | 7.081 | <0.001 | 0.350 |
| NfL ~ sex(male) | -16.008 | 6.955 | -2.302 | 0.021 | -0.114 |
|  |  |  |  |  |  |
| **Defined (Indirect / Total Effects)** |  |  |  |  |  |
| Indirect effect PC1  (a1 × b1) | -0.087 | 0.038 | -2.270 | 0.023 | -0.027 |
| Indirect effect PC2  (a2 × b1) | -0.059 | 0.034 | -1.765 | 0.078 | -0.018 |
| Total effect PC1 | -0.087 | 0.038 | -2.270 | 0.023 | -0.027 |
| Total effect PC2 | -1.053 | 0.161 | -6.554 | <0.001 | -0.324 |
